# The value of radiographic indexes in the diagnosis of discogenic low back pain: a retrospective analysis of imaging results

**DOI:** 10.18632/oncotarget.18652

**Published:** 2017-06-27

**Authors:** Jian Song, Hong-Li Wang, Xiao-Sheng Ma, Xin-Lei Xia, Fei-Zhou Lu, Chao-Jun Zheng, Jian-Yuan Jiang

**Affiliations:** ^1^ Department of Orthopaedics, Huashan Hospital, Fudan University, Shanghai, 200040, China

**Keywords:** discogenic low back pain, angular motion, modic change, HIZ

## Abstract

To explore value of different radiographic indexes in the diagnosis of discogenic low back pain (LBP). A total number of 120 cases (60 patients diagnosed with discogenic LBP and 60 healthy people) were retrospectively analysed to identify factors in the diagnosis of discogenic LBP by using univariate and multivariate analyses. A receiver operating characteristic (ROC) curve was drew to show the predictive accuracy of the finally enrolled factors. Among all the included patients, 60 were strictly admitted in the discogenic LBP group while the other 60 were enrolled in the control group. Five results shows significant differences between discogenic LBP and control groups, including Cobb angle, lumbar stability, height of the disc, Modic change and High intense zone (HIZ) based on the results of univariate analysis; lumbar stability, Modic change and HIZ show high value in the diagnosis of lumbar discogenic pain based on the multivariate logistic analysis. The ROC curve shows that good diagnostic accuracy was obtained from the enrolled diagnostic factors including lumbar stability (Angular motion, more than 14.35°), Modic change and HIZ.

## INTRODUCTION

LBP has been considered to be the top leading cause for years lived with disability globally, while lumbar discogenic pain is the main cause of LBP [1–3]. Report has confirmed that discogenic LBP is due to degenerative disc disease (DDD) by the magnetic resonance (MR), and the disease can be relieved by the injection of contrast media or local anesthesia into the disc [4–6]. There are a lot of factors which are correlated to the discogenic LBP, such as lumbar spine stability, degree of lumbar intervertebral disc (IVDs) degeneration and pathobiology of Modic changes [7–8]. Recently, studies using animal discogenic pain models and specimens from degenerated human IVDs have provided insights into the pathomechanisms of discogenic LBP, some one believed that painful discs are characterized by a confluence of innervation, inflammation factors [9–10]. Despite its enduring presence, pain ostensibly emanating from a disc itself has hitherto remained poorly defined, and its diagnosis factors have been exceedingly controversial.

Spinal imaging modalities have been widely used to diagnose discogenic LBP, including MR, computed tomography (CT), plain radiography (X-rays), myelography, and CT-myelography, while provocative discographys is the gold standard [11–13]. The most frequent imaging modality was MR, followed by X-rays and CT, while Myelography and CT-myelography alone were scarcely used [5]. However, analysis of imaging measurement in the diagnosis of discogenic LBP still remains absent. Thus, we carried out this research aiming to characterize a carefully selected cohort of the patients with discogenic LBP and to elucidate the factors in the diagnosis of discogenic LBP by using univariate and mulitivariate analyses.

## RESULTS

### Characteristics of enrolled patients

**Figure 1:**
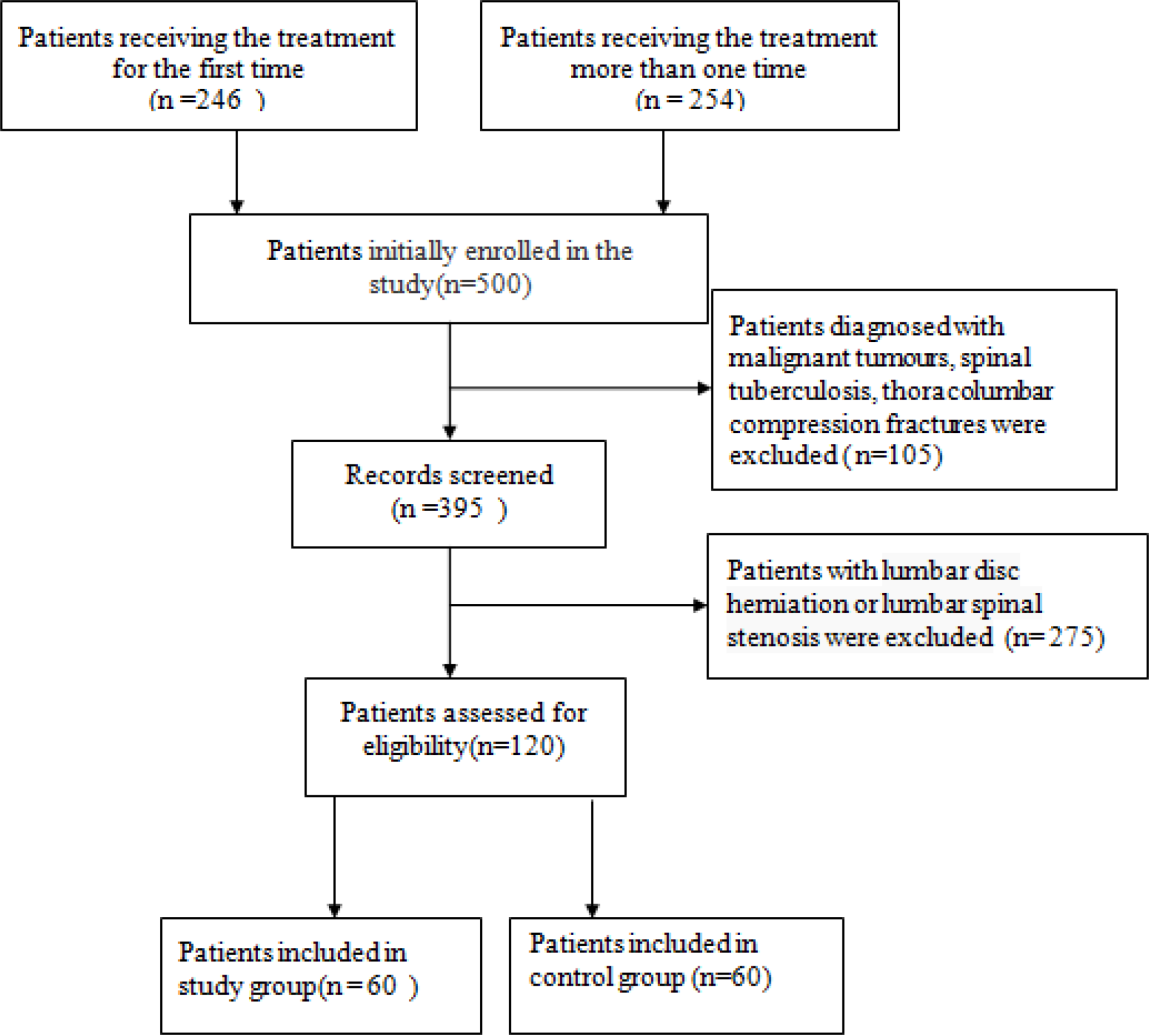
Flow diagram of this population-based study

500 patients diagnosed with LBP were primarily admitted to our study between October 2007 to December 2016 from our hospital. The age of the patients ranged from 30 to 70 years old. We excluded the patients from the studies if they were diagnosed with malignant tumors, spinal tuberculosis, thoracolumbar compression fractures (*n* = 105). A total number of 395 patients were left after this screening. Patients with lumbar disc herniation or lumbar spinal stenosis were also excluded (*n* = 275). 120 patients were assessed for eligibility finally. 60 patients with positive results of provocation discography were selected as study group and 60 patients with negative results of provocation discography were regarded as control group (Figure 1). All the characteristics and radiographic results of enrolled patients were specifically listed in the Table 1.

**Table 1:** Characteristics of enrolled discogenic LBP and control group

Characteristics	Discogenic LBP (*n* = 60)	Control group (*n* = 60)	Value of χ^2^ or t	*P*
Age of the patients(years)	50.58 ± 11.02	50.25 ± 12.09	0.25	0.563
Gender				
Male	33 (55.0%)	35 (58.4%)		
Female	27 (45.0%)	25 (41.6%)	0.14	0.713
Disc position				
L3-4	6 (10.0%)	8 (13.3%)	0.32	0.570
L4-5	37 (56.7%)	39 (65%)	0.14	0.705
L5-S1	17 (28.3%)	13 (21.7%)	0.71	0.399
Total			0.87	0.643
Cobb angle of Lumbar vertebra (T12-S1) (°)	3.73 ± 2.24	2.93 ± 1.91	2.11	0.040
Lumbar lordosis angle (L1-S1) (°)	45.3 ± 8.6	43.6 ±7.4	1.20	0.100
Lumbar stability (lesion segment)				
Angular motion (°)	19.09 ± 3.44	10.33 ± 3.02	14.85	0.000
Lumbar mobility (mm)	4.94 ± 3.18	2.19 ± 2.35	5.35	0.000
Height of the disc (mm)				
Anterior	12.68 ± 1.99	15.67 ± 2.63	−4.96	0.022
Middle	10.88 ± 2.22	13.22 ± 1.70	6.19	0.016
Posterior	9.38 ± 2.13	10.78 ± 1.29	−4.23	0.028
Spinal canal dimensions(mm)				
Sagittal diameter	13.98 ± 2.25	14.09 ± 3.33	−2.01	0.054
Tranverse diameter	23.26 ± 3.77	24.51 ± 3.20	−1.96	0.063
VAS score	7.6 ± 2.3	7.2 ± 1.6	3.2	0.143
Modic change				
Negative	5 (8.3%)	55 (91.7%)	33.75	
I	17 (28.3%)	5 (8.3%)	22.34	
II	31 (51.7%)	0 (0%)	51.79	
III	7 (11.7%)	0 (0%)	7.43	
Total			62.02	0.000
HIZ				
Positive	31(51.7%)	7 (11.7%)		
Negative	29(48.3%)	53 (88.3%)	22.18	0.000

### Results of the univariate analysis

Univariate analysis was carried out by using student's *t*-test (continuous data) and the χ^2^ test (dichotomous data). All the results including the basic characteristics and radiographic measurement were fully showed in the Table 1. The control group owned smaller Cobb angle when compared with the discogenic LBP group (2.93 ± 1.91 vs 3.73 ± 2.24; *p* = 0.04). The patients with discogenic LBP owns lower lumbar stability (Angular motion, lumbar mobility) when compared with the control group(19.09 ± 3.44 vs 10.33 ± 3.02, *p <* 0.001; 4.94 ± 3.18 vs 2.19 ± 2.35, *p <* 0.001). The height of the lesion disc in the discogenic LBP group is much lower than the control group (anterior, middle, posterior) (12.68 ± 1.99 vs 15.67 ± 2.63, *p* = 0.022; 10.88 ± 2.22 vs 13.22 ± 1.70, *p* = 0.016; 9.38 ± 2.13 vs 10.78 ± 1.29, *p* = 0.028). More Modic changes and HIZ happened in the discogenic LBP group when compared with the control group (55:5 vs 25:35, *p* < 0.001; 31:29 vs 7:53 *p* < 0.001).

### Results of multivariate logistic regression

**Figure 2:**
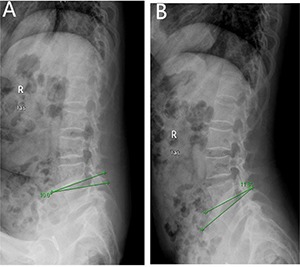
Angular motion of a 48 years old man,diagnosed with discogenic low back pain (LBP) for 2 years (**A**) delineates flexion angle measured by X-ray (L4-5). (**B**) indicates the extension angle measured by X-ray (L4-5).

**Figure 3:**
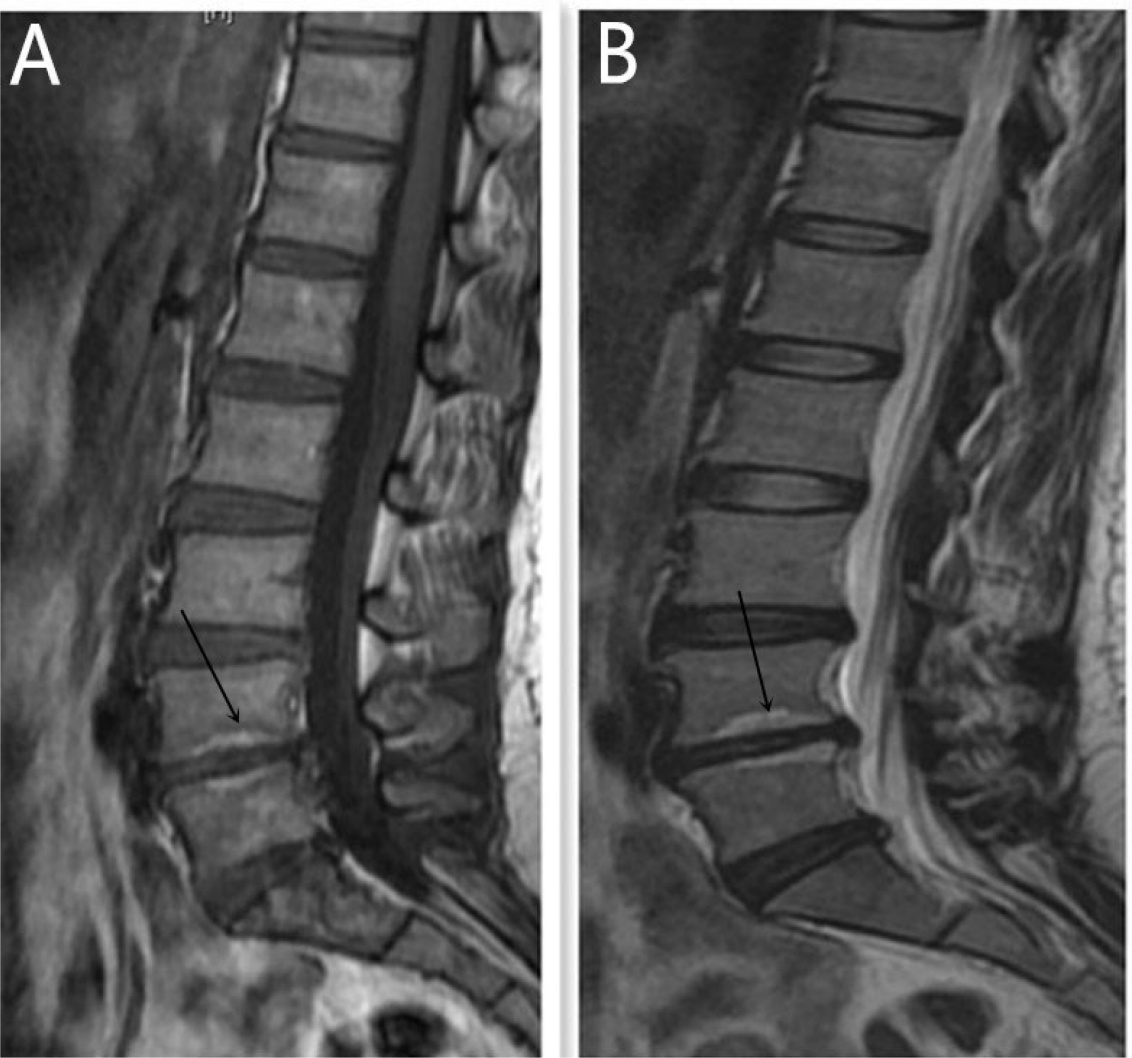
Modic type 2 changes was presented by MR for a 46 year old women, diagnosed with discogenic LBP for 10 months (**A**) is High T1 signal (L4-5). (**B**) indicates High T2 signal (L4-5).

**Figure 4:**
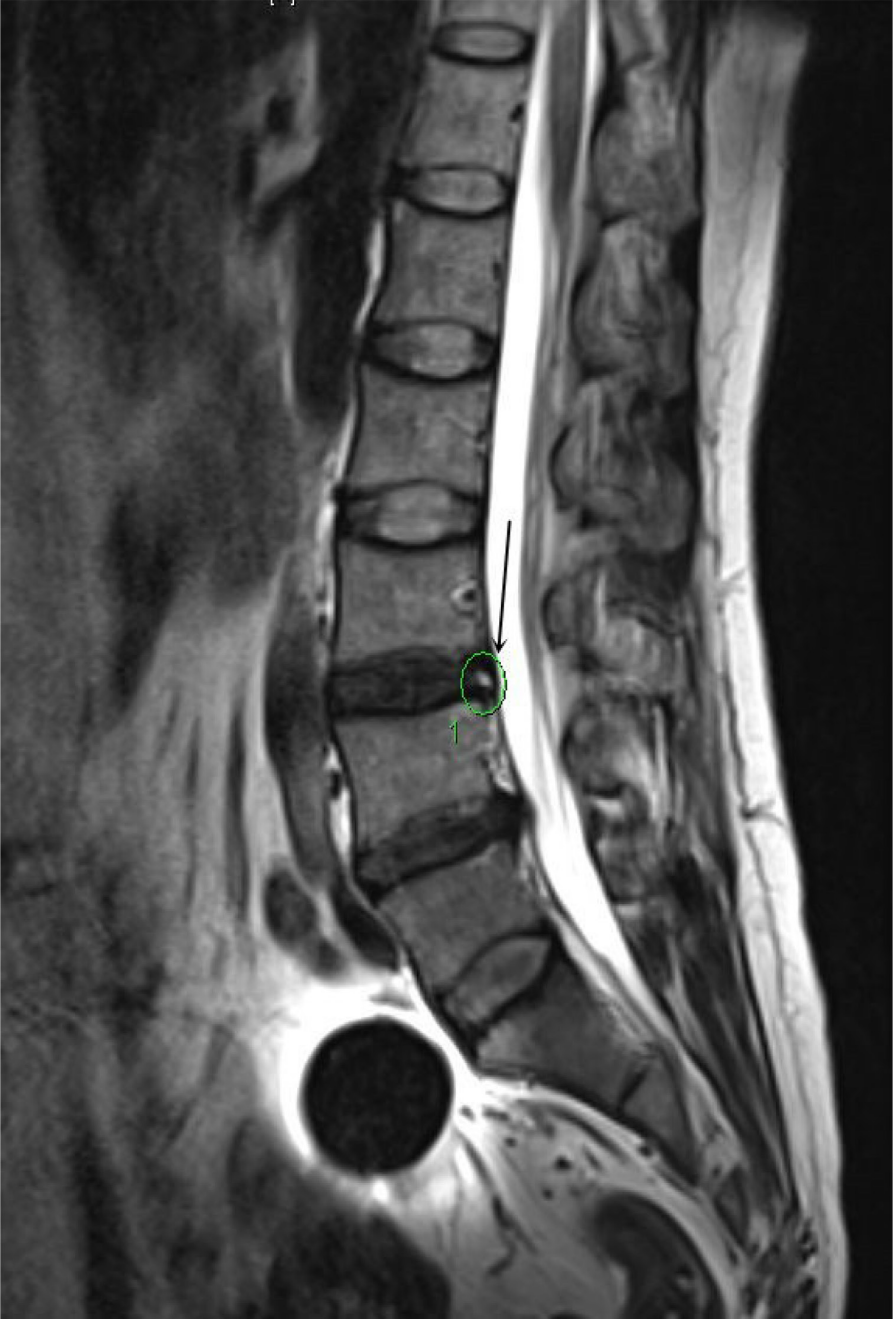
HIZ was presented by MR at the T2 signal (L3-4) for a 56 years old women, diagnosed with discogenic LBP for 3 years

All statistically significant (*P* < 0.05) covariates based on the univariate analysis, including age of the patients, Cobb angle, lumbar stability, height of the disc, Modic change and HIZ, were subjected to multivariate logistics regression analysis. The results showed that Angular motion, Modic change and HIZ were independently associated with discogenic LBP (*p* = 0.001; *p* = 0.017; *p* = 0.013) (Table 2) (Figures 2, 3, 4).

**Table 2:** Logistic regression of risk factors for discogenic low back pain

Factors	B	S.E.	Wald	*P*	95%CI
Angular motion	0.103	0.044	5.403	0.001	0.107∼0.576
Modic change	−2.688	1.129	5.673	0.017	0.007∼0.621
HIZ	−5.976	2.402	6.190	0.013	0.000∼0.281
Constant	23.565	7.202	10.711	0.001	—

B: regression coefficient; S.E: standard error

### Predictive accuracy of the factors in the diagnosis of discogenic LBP

**Figure 5:**
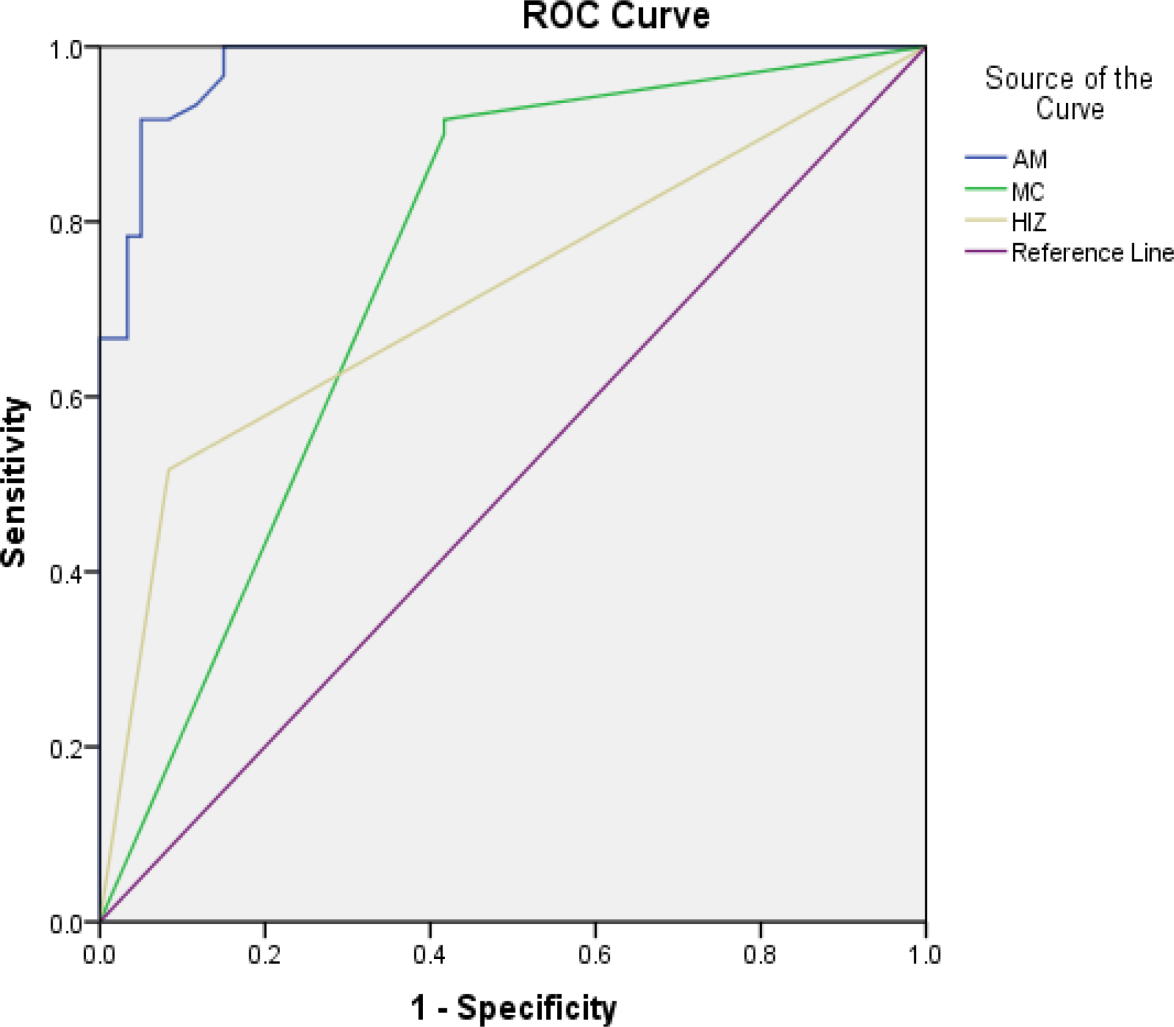
The ROC curve for the diagnosis of discogenic LBP AM: Angular motion, MC: Modic change, HIZ: high intense zone.

A ROC curve was drew to show predictive accuracy of the enrolled factors. The area under the curve of each enrolled factor is more than 0.5, which were set as reference line, indicating good diagnostic accuracy of the enrolled factors. Angular motion 0.978 (more than 14.35°); Modic change 0.747; HIZ 0.717 (Figure 5).

## DISCUSSION

It is not easy to use the symptoms and clinical examinations to diagnose or localize the level of abnormality as the discogenic LBP has a somatotropic rather than dermatomal pattern of pain projection [9]. Several other modalities have been used to help the diagnosis of discogenic LBP, such as MR, CT, myelography, and X-ray [5]. The most frequent imaging modality was MR, which could provides a unique means to evaluate the morphologic status of discs. Study show that MR showed a significant correlation with the discography findings in evaluating disc degeneration as most of the discs with normal morphology on MR were also normal on discography [13]. The clinical relevance of a posterior HIZ on T2-weighted MR images represents fluid or mucoid material associated with granulation tissue and new blood vessels entrapped between torn fibers of the outer annulus or underneath the posterior longitudinal ligament complex [14]. HIZ is mainly due to mechanical fatigue loading, and inflammatory reactions associated with repair of an annular tear. For many patients, the inflammatory mediators such as TNR-α, IL-1, IL-6 released following the annular fissures [15–16] may explain the LBP and their high signal intensity on T2-weighted images. According to the results of the univariate analysis, 31 patients (51.7%) show posterior HIZ on T2-weighted MR images in the study group while only 7 patients (11.7%) in the control group show the same appearance, significant difference was detected between the two groups (t = 22.1; *p* = 0.00). We also confirm that high diagnostic value of HIZ were also obtained based on the results of multivariate analysis (*p* = 0.013) and ROC curve(AUC=0.717). So we conclude that HIZ on T2-weighted MR images is credible to forecast the discogenic LBP.

Modic changes appear as the subchondral signal abnormalities on MRI in the bone marrow adjacent to degenerated discs, and it has also be confirmed to be associated with discogenic LBP [17–19]. An recent cadaver research showed that bony endplate lesions had association with the history of LBP [20], and both Modic changes and bony endplate defects could contribute to the LBP. Increased signal intensity (SI) of nucleus pulposus and an accelerated process of disc degeneration have also been confirmed in the process of Modic changes [21–22]. More patients in the symptomatic group own Modic change when compared with the control group (I,17 vs 5; II,31vs 0;III,7 vs 0; *P* = 0.00) based on the results of the univariate analysis. Modic changes can also applied to forecast the discogenic LBP and show high diagnostic significance according to the results of multivariate analysis (*p* = 0.017) and ROC curve (AUC = 0.747). The results conducted by our research confirmed the association between the discogenic LBP and Modic change.

X-ray is also widely used to help the diagnosis of discogenic LBP as the angle motion, defined as difference of the flexion and extension angle, can describe degree of the hypermobility. Hypermobility is also thought to play an important role in the IVDs degeneration as IVDs bears major load in humans [23]. Discogenic LBP is mainly due to IVDs degeneration and such process is defined by changes in architecture and biochemical composition that invariably alter the internal mechanical environment of the disc. The anular fibers become torn and disorganized, the nucleus becomes less hydrated and it is difficult to distinguish the border between the anulus and nucleus [10]. Those changes contribute to altering the constraints placed on adjacent vertebrae, leading to spinal hypermobility. Such type of mechanical exposure initiates damage from the outside and then works inward via anular delamination and disruption, cellular metaplasia, and vertebral rim hypertrophy as anular fibers is the peripheral disc tissue that acts to restrict intervertebral movement [24]. Ariga et al. [25] have also linked hypermobility to the development of cellular apoptosis and matrix damage within the cartilage end plate by using a mouse mode. The results show that patients in the study group owns much more Angle motion when compared with the patients in the control group (19.09 ± 3.44 vs 10.33 ± 3.02; *P* = 0.00), and the it is also the factor showing high diagnostic significance of the lumbar discogenic pain based on the multivariate analysis (*p* = 0.020) and ROC curve (AUC = 0.978).

Discography, regarded as a pain provocation test, has been considered to be the gold standard in the diagnosis of discogenic LBP and it is also the only method that could be applied to relate a radiologic image to the patient's pain directly [26]. The concordant pain at the adjacent disc levels happen in some cases when provocative discographys were carried out for the patients suspected with discogenic LBP, some were two level lesions, while some others were only one diseased level. According to the diagnostic criteria of discogenic LBP, the lack of concordant pain response at adjacent normal appearing disc levels was required. In order to select those patients with discogenic LBP and position the diseased level correctly, we included those patients with only one lesion level.

Another three results are considered to be associated with discogenic LBP, including age of the patients, Cobb angle, height of the disc; however, they could not be applied to forecast such disease. Discogenic LBP is mainly due to IVDs degeneration, so we hypothesize that it is associated with the increasing age and the result confirmed that based on the univariate analysis [27]. The Cobb angle has become the parameter for quantifying scoliosis curve magnitude. Long term of scoliosis may aggravate local compression and degeneration of the lumbar IVDs. A study has been carried out indicating that LBP was correlated to scoliosis [28]. Research shows that gender (Female), LBP at baseline, radiographic knee osteoarthritis were associated with increased risk for disc height narrowing [29]. IVDs degeneration is associated with progressive morphological, structural, histological, biochemical and functional changes. Severe morphological changes, disc prolapse and end-plate damage, such as anulus tears, are obviously seen with the progression of the disc degeneration. At the stage, disc height narrowing becomes obvious on lumbar radiographs.

Some limitation may exist in the present study. First, this is a retrospective study, as in so many similar published study, may induce section bias. Secondly, the duration of follow-up varied considerably as the enrollment period was so long. Thirdly, the results of univariate and multivariate analyses may be different from the real results because of the limited number of enrolled patients.

## MATERIALS AND METHODS

### Ethical considerations

The study protocol was approved by the ethics committee of Huashan hosital, Fudan university. All the informed consent from the enrolled patients has been acquired as it was a retrospective study. All the methods carried out in the research were performed in accordance with the relevant guidelines and regulations.

### Patients enrolling and diagnostic criteria of discogenic LBP

**Figure 6:**
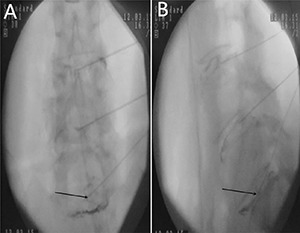
Provocation discography for a 54 years old woman, who had sufferedfrom low back pain (LBP) for more than 2 years The results shows the ruptured intervertebral disc (L5-S1), leakage of the contrast medium, concordant pain (7 point on the numeric rating scale) was induced according to the VAS score. (**A**) is the frontal X-ray of the provocation discography. (**B**) indicates the lateral X-ray of the provocative discography.

Patients diagnosed with discogenic LBP were admitted in our study from the department of orthopaedic in our hospital between October 2007 to December 2016; The patients enrolled in the study and control groups should fulfill all of the diagnostic criteria described below: (1) The age of the patients ranged from 30 to 70 years olds. (2) The patients suffered from discontinuous LBP more than 6 months without traumatic history. (3) T2-weighted Magnetic resonance (MR) shows the degenerative intervertebral disc low signal. (4) Concordant pain were recorded during the provocative discography,the presence of a pain response on the Visual Analogue Scale (VAS) of was 6 or higher, adjacent discs show normal appearance or subtle degeneration (one abnormal disc with a normal appearing disc above and below) [30] (5) The level of pfirrman grade is not less than III based on the T2-weighted sagittal images [31]. (6) Only one diseased intervertebral disc is involved (Figure 6). Patients were excluded from the study if they also suffered from malignant tumors, spinal tuberculosis, thoracolumbar compression fractures or some other diseases that could cause LBP (lumbar disc herniation or lumbar spinal stenosis). The same amount of patients with LBP, who own negative results of provocative discography,were enrolled as the control group. For the patients with negative results of provocative discography, two-year follow-up results show that LBP disappears. We also excluded those patients, whose pfirrman grade level was more than III based on the T2-weighted sagittal images in the control group.

### Radiographic measurement and analyses of the factors

Angle motion of the lumbar spine intervertebral was measured to indicate the degree of spine instability and include angle motion and lumbar mobility [32]. Two lines across the upper and lower vertebral body margin of lesion level insect and form the angle at flexion and extension position by X-ray (Phillips, Eindhoven, Netherlands), difference of the flexion and extension angle was defined as the angle motion of lumbar spine [33]. Lumbar mobility was measured by calculating the distance between upper and lower vertebral body of the lesion level at the lateral position by X-ray. The cobb angle of interest is simply the angle between the two line drew from the upper endplate of the upper body and along the lower endplate of the lower body [34]. Height of the lesion disc was measured by supine MR (Siemens, Germany). The T1 and T2 intensity images were constructed with the TE/TR of 10/500 ms and 100/2800 ms. The slice thickness was 4 mm. We performed the measurement on the T2 intensity images at the sagittal planes. The measurement of the vertical intervertebral disc lengths was performed on the mid-saggital section of the vertebral body (anterior, middle and posterior). Measurement of spinal canal dimensions of the lesion level (saggital diameter and transverse diameter) was carried out by supine MR to reflect degree of the lumbar spinal stenosis [35]. HIZ was defined as a small, round zone with limited high-intensity signals in the posterior annulus of lumbar intervertebral discs on sagittal slices of T2-weighted MR, it also represents a deep radial fissuring the annulus fibrosis of the lesion level, just as revealed by lumbar CT discography [36–39]. Modic changes are usually displayed by MR to describe the signal intensity changes of vertebral end-plate of the lesion disc [40]. Modic type 1 changes (MC1) refers to low T1 and high T2 signal, MC2 refers to high T1 and T2 signal, and MC3 refers to low T1 and T2 signal [41]. Grading of disc degeneration was assessed from T2-weighted sagittal images based on the Pfirrmann method [31]. Two independent spine surgeons performed the measurement by using Centrieity Enterprise Web V3.0 (General Electric, US).

### Statistical analysis

We used SPSS 21.0 (SPSS, Chicago, IL, USA) to perform all the statistical analyses. Dichotomous data was described as counts and percentages while continuous data are presented as means ± standard deviation. Univariate analysis was carried out by using student's *t*-test(continuous data) and the χ^2^ test (dichotomous data). All the performed statistical tests were two-tailed. The statistically significant (*P* < 0.05) covariates based on the univariate analysis were subjected to multivariate logistics regression analysis. A receiver operating characteristic (ROC) curve was drew to show the predictive accuracy while the value of sensibility was set up as vertical coordinate and the value of 1- specificity was set up as the horizontal ordinate. A two-tailed *P*-value < 0.05 was considered to be statistically significant between the two groups.

## CONCLUSIONS

Lumbar stability (Angular motion, more than 14.35°), Modic change and HIZ show high diagnostic value in the diagnosis of discogenic LBP based on the multivariate analysis, good diagnostic accuracy was obtained according to the ROC curve.
